# Purification and characterisation of a sulphur rich melanin from edible mushroom *Termitomyces albuminosus* Heim

**DOI:** 10.1080/21501203.2018.1494060

**Published:** 2018-07-09

**Authors:** Rosy Agnes De Souza, Nandkumar Mukund Kamat, Vishnu S. Nadkarni

**Affiliations:** a Mycological Laboratory, Department of Botany, Goa University, Taleigao, Goa, India; b Department of Chemistry, Goa University, Taleigao, Goa, India

**Keywords:** *Termitomyces*, melanin, submerged fermentation, DOPA, purification

## Abstract

Production, purification and characterisation of a black pigment from *Termitomyces albuminosus* as melanin is reported, for the first time, from shaken submerged culture condition using scanning electron microscopy (SEM), elemental analysis, ultraviolet–visible (UV-VIS), and Fourier transformed infrared spectroscopy (FTIR), electron paramagnetic resonance (EPR) and ^13^C (CP/MAS) NMR spectra. SEM results on *T. albuminosus* revealed nanogranular nature of melanin nanoparticles within size range of 400–100 nm with fractal dimension *D* = 1.195–1.73. Elemental analysis of melanin indicated 54.6% C, 3.5% H, 2.4% N, 26.9% O, and 12% S. UV-VIS and FTIR spectra confirmed to the characteristic of melanin and were identical to the reference commercial sepia melanin. Further validation of the identity of pigment as melanin was achieved by EPR analysis. *Termitomyces albuminosus* melanin is postulated to be DOPA-type melanin confirmed by ^13^C (CP/MAS) NMR spectral analysis showing chemical shift at 200–170 ppm carbonyl, 160–110 ppm aromatic region, and with high 40–30 ppm open chain aliphatic region. Chemical modification through oxidation and cysteinylation (Pheomelanin) is implied as indicated by relatively high sulphur content (12%).

## Introduction

Melanin biosynthesis is a common feature in kingdom fungi. The pigment not essential for hyphal growth appears as secondary metabolite. Melanins are most stable, amorphous polymers of phenolic compounds and can be classified into the following three types: eumelanins, pheomelanins and allomelanins. Melanin production helps in protection from extreme environmental conditions such as UV light, ionising radiation, resistance to heat or cold, phagocytosis, heavy metals, and oxidants and provides cell wall rigidity (Money et al. 1998; Plonka and Grabacka 2006; Pal et al. 2013; Casadevall et al. 2017). Despite its importance and ubiquity, many fundamental questions remain unanswered like details of its chemical structure and insolubility (Eisenman and Casadevall 2012). Some fungi undergo melanogenesis in response to certain environmental stress conditions such as extreme temperatures, dessiccation, hyperosmotic conditions, limited nutrients, pH changes, metal toxcicity, UV or ionisation stress, action of antagonistic microbes. Melanisation in fungi mostly seen in hyaline hyphae, sclerotia, appressoria, reproductive structures or conidia (Cordero and Casadevall 2017). Hyphal melanin is often found to be deposited as the outermost layer or internal layer in cell wall only with age or other stress (Bell and Wheeler 1986; Henson et al. 1999; Butler et al. 2001). Melanogenesis in pathogenic fungi plays a key role in pathogenesis in species such as *Cryptococcus neoformans* (Polacheck and Kwon-Chung 1988), *Gaeumannomyces graminis* var. *tritici, Magnaporthe grisea, Alternaria alternata, Colletotrichum lagenarium, Cochliobolus heterostrophus* (Henson et al. 1999), *Paecilomyces variotti* (Babitskaya et al. 2000a), *Rhizoctonia solani* (Chen et al. 2015) and *Aspergillus* spp. (Babitskaya et al. 2000a; Schmaler-Ripcke et al. 2009; Gonçalves et al. 2012; Pal et al. 2013). Melanins are reported from mushrooms such as *Agaricus bisporus* (Mendoza et al. 1979), *Inonotus obliquus* (Babitskaia et al. 2000b; Babitskaya et al. 2002), and *Schizophyllum commune* (Arun et al. 2015). Plant-associated symbiotic ectomycorrhizal fungus, *Cenococcum geophilum*, produces melanin under dehydrated conditions (Fernandez and Koide 2013). Fungi synthesise melanin by one of the two synthetic pathways: 1,8-dihydroxynaphthalene (DHN) intermediate and l-3,4-dihydroxyphenylanine (L-DOPA). Melanin synthesis involves copper containing metalloenzymes such as laccase and tyrosinase and in fungi also shows involvement of chitin cross-links to other cell wall polysaccharides and proteins (Eisenman and Casadevall 2012). Studies on melanins in mushrooms are limited to edible mushrooms such as *Pleurotus cystidious* var. *formosensis, P. australis*, and *P. purpureoolivaceus* from which darkly pigmented arthroconidia forming black pigment on mycelium or basidiomata has been characterised (Selvakumar et al. 2008). According to Mendoza et al. (1979), the spore wall of *Agaricus bisporous* and *Agaricus campestris* contain 26–28% and 24–26% crude (dry weright cell wall) melanin. Mushroom fruitbody decolourisation is very common due to oxidation of phenolic substrates into quinones leading to the formation of brown-coloured melanin in species such as *A. bisporous*, thus decreasing its commercial value (Weijn et al. 2013). Exo- and endomelanin complex of *Inonotus obliquus* and *Phellinus robustus* in submerged conditions demonstrate high-antioxidant and genoprotective properties (Bisko et al. 2002, 2007). Melanin in *Auricularia auricula* has been studied extensively (Zou et al. 2010; Bin et al. 2012; Zhang et al. 2015; Sun et al. 2016a). Melanin is found useful in the field of material science as coating material in electronic/bioelectronics, drug delivery and cosmetics as sunscreens, emphasising the importance of finding good, non-toxic melanin sources (Blumenberg 2017).

Symbiotic fungal species in *Termitomyces* Heim are found in Asian and African continents as exosymbionts cultivated by fungus growing termites belonging to subfamily – *Macrotermitinae* in their nest as food (Wood and Sands 1978). During tropical monsoon, fruitbodies from subterranean fungus combs emerge by forcing their way through very hard layer of inert matter using a hard, melanised perforatorium (Heim 1977; Kendrick 2001). Traditionally, these species are known to be most popular and highly prized edible mushrooms in Asia and Africa. Taxonomists have reported dark pigmentation in fruitbodies especially in organs like hypogeal pseudorrhiza and epigeal smooth or pointed umbo exhibiting brownish to greyish-black colouration, without commenting on chemical nature and role of such dark pigmentation, thus leaving the issue of its chemical identification and characterisation open (Otieno 1968; Pegler and Rayner 1969, 1969; Natarajan 1979; Van Der Westhuizen and Eicker 1990; Pegler and Vanhaecke 1994; Abdullah and Rusea 2009; De Kesel 2011; Srivastava et al. 2011; Tibuhwa 2012; Karun and Sridhar 2013; Aryal et al. 2016).

In spite of extraction of melanin from several edible mushroom species, there is no knowledge regarding edible melanin obtained from a symbiotic mushroom which can provide better source of mushroom melanin as this *Termitomyces* species is well consumed in entire Asian and African continent for its delicacy. The present study thus aimed to produce the dark melanin-like pigment from pure culture under controlled conditions, purify it and verify its chemical identity as melanin and characterise it structurally.

## Materials and methods

### Source and growth conditions of melanic culture

Fresh, healthy *Termitomyces albuminosus* fruitbodies were collected from Mardol, Goa during monsoon season and taxonomically identified using standard published *Termitomyces* keys (Heim 1942, 1977). Several pure cultures were obtained from sterile context tissue explants of pileus on 2% Malt Extract Agar (MEA) medium (Malt extract refined bacteriological grade 2% and Agar bacteriological grade 2%) with 0.01 mg/mL concentration of nalidixic acid and neomycin (HiMedia Chemicals Ltd., Mumbai, India). Growth, morphology, and pigmentation in colonies were monitored and a promising strain showing dark melanin like pigmentation was selected and microscopically checked for purity. The melanic strain was deposited in Goa University Fungus Culture Collection (WFCC Reg. no. 946) bearing GUFCC No. 20002 and maintained on Czapek Dox Agar (CDA) medium (0.5% sucrose, 0.2% sodium nitrate, 0.1% dipotassium phosphate, 0.05% magnesium sulphate heptahydrate, 0.05% potassium chloride, 0.001% ferrous sulphate heptahydrate, and 2% agar bacteriological grade), pH 5.5 and was incubated in incubator (Modern Industrial Corporation, Mumbai, India) at 28 ± 1 °C in dark.

### Production of melanin in shaken submerged culture condition

Ten identical culture plugs were inoculated into 250 mL Erlenmeyer flasks containing 100 mL of Czapek Dox Solution (CDS) and were incubated on rotary shaker (Scigenics Biotech, Orbitek model LETT-A, Tamil Nadu, India) at 28 ± 1 °C, pH 5.5 for 1 week in dark with shaking at 150 rpm. Mycelial suspensions were obtained from pellets (Kalisz et al. 1986). Inoculum (10% v/v) was transferred into 2000 mL Erlenmeyer flasks containing 1000 mL of CDS having 5 g/L sucrose, pH 5.5 and incubated at 28 ± 1 °C for 20 days on rotary shaker at 150 rpm. Flasks were incubated at room temperature for 20 days. Insoluble melanin bound to mycelial biomass was extracted after 20 days.

### Melanin extraction and purification

*Termitomyces albuminosus* pellet biomass was harvested using sterile stainless steel sieve of 100 µm mesh size, washed with sterile double distilled water three times, and oven dried at 70 °C overnight to a constant weight for estimation of mycelial dry weight. Melanin was extracted from the dry powdered fungal biomass using modification in previously described method (Sun et al. 2016b). Dry biomass powdered using mortar and pestle was subjected for melanin extraction in 100 mL 2 mol/L NaOH, in autoclave at 120 °C for 20 min. Extracts obtained were centrifuged at 5000 rpm for 5 min., supernatant was adjusted to pH 1.5 with 7 mol/L HCl, then kept at room temperature (RT) for 2 h and centrifuged at 8000 rpm for 20 min to collect the precipitate. The precipitate was washed three times with milliQ water, and dried and redisloved in 2 mol/L NaOH and surpernatent was collected after centrifugation at 8000 for 20 min. The supernatent pH was readjusted to pH 1.5 with 7 mol/L HCl and then kept at RT for 2 h. The precipitate was collected by centrifugation at 8000 rpm for 20 min. The precipitates obtained of crude mealnin were hydrolysed with 7 mol/L HCl at 100 °C for 2 h in order to remove bound carbohydrates and proteins. Then contents were cooled at RT and precipitate was collected by centrifugation at 8000 rpm for 20 min. The precipitate was washed three times with milliQ water to remove chloridion followed by drying at RT. The dried melanin was sequentially washed with chloroform, ethyl acetate and absolute ethanol in order to remove bound lipids, dried at RT and was transferred to a desiccator. Subsequently, the dried melanin was redissolved in 2.0 mol/L NaOH, followed by centrifugation at 8000 rpm for 20 min. The supernatant was adjusted to pH 1.5 and centrifuged at 8000 rpm for 20 min. The pure melanin was obtained after repeated washing of the precipitate with milliQ water and then drying to a constant weight in an oven at 60 °C. Purified melanin was stored in an air tight, moisture free amber bottle at −20 °C.

### Morphology of melanin particles

#### Bright field microscopy

Culture from dark pigmented colonies of *T. albuminosus* and smaller melanised pellets were mounted in plain lactophenol. Pure melanin particles obtained by purification process were mounted in DPX on slides and examined using Nikon Eclipse E200 microscope with Nikon DS-fi2 camera and NIS element microscope imaging software.

#### Scanning electro microscopy (SEM)

Pure dried powdered melanin particles were fixed on carbon tape on aluminium stub and sputter coated with Palladium for 10 s (Quorum SC7620 Sputter Coater, UK) and examined by SEM at 5 kV (Vega 3 SB, TeScan, Advanced Scientific Equipment Pvt. Ltd., Bangalore, India).

#### Fractal analysis

SEM images of 10000× magnification were subjected to 11 different mathematical methods to compute fractal dimension using CMEIAS JFrad version 1.0 software freely available at http://cme.msu.edu/cmeias/ (Ji et al. 2015). The output data of melanin fractal dimensions were saved as *csv files and analysed statistically using the SYSTAT 13.

### Elemental composition of melanin

The elemental composition CHN (O) of pure *T. albuminosus* melanin was determined with approximately 5 mg solid samples using elemental analyser (Thermo Finnigan, Italy model FLASH EA 1112 series, SAIF–IIT Bombay analytical laboratory, India) dispersed in water. The sulphur content was computed after addition of C, H, N, O percentages and qualitatively detected using Lassaigne’s test (Harki et al. 1997).

### Ultraviolet–visible (UV-VIS) and Fourier transform infrared spectroscopy (FTIR)

UV-VIS spectrum was obtained in the range 190–750 nm using UV–VIS spectrophotometer (Shimadzu UV-2400) 0.1 mol/L NaOH as reference (Suryanarayanan et al. 2004; Selvakumar et al. 2008). A standard melanin spectrum was also obtained using *Sepia officinalis* melanin (Sigma, Aldrich Chemicals, India). For FTIR spectral analysis, the purified *T. albuminosus* melanin sample was mixed with KBr (1:10) and pressed into a 1 mm thin pellets. FTIR spectra were recorded between 4000 and 500 cm^−1^ in transmission/absorbance mode on FTIR spectrometer (Shimadzu IR Prestige 21, Japan) averaging of 40 scans. Spectral resolution was 4 cm^−1^, encoding interval 1 cm^−1^, Happ–Genzel apodisation and scanning speed 2.8 mm s^−1^ (Mbonyiryivuze et al. 2015).

### Electron paramagnetic resonance (EPR) spectroscopy

EPR spectra were recorded using 25 mg samples at 77 K using ESR–JEOL, Japan model JES–FA200 ESR spectrometer for x band (SAIF–IIT Bombay analytical laboratory, India). Parameters used to acquire the spectra were as follows: modulation amplitude, 0.16 mT; modulation frequency 100 KHz; centre field, 325 mT; sweep width, 25 mT; sweep time, 2 min; microwave frequency, 9.1 GHz; microwave power, 0.1 mW; and temperature 77 K (Enochs et al. 1993).

### NMR studies

Solid-state ^13^C (CP/MAS) NMR spectra were acquired on a Bruker Avance II 500 MHz spectrometer at Central Salt and Marine Chemicals Research Institute (CSMCRI) analytical laboratory, India.

## Results

### Cultural growth and melanin production

**Figure 1.**
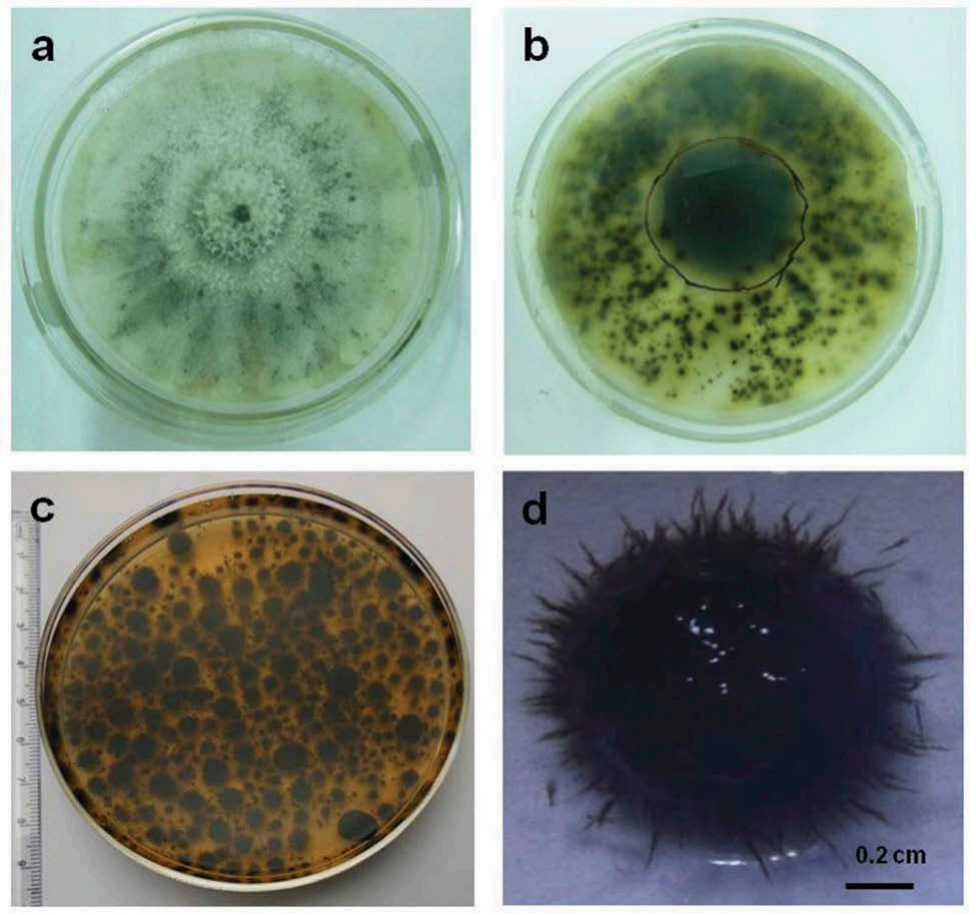
Melanin production in *Termitomyces albuminosus* Culture. (a) *T. albuminosus* colony surface view. (b) *T. albuminosus* colony reverse view. (c) *T. albuminosus* pellets production in submerged shaken condition. (d) Single-pellet morphology.

*Termitomyces albuminosus* colonies on CDA after 8 days showed 7.9 ± 0.17 cm diameter, initially cottony white but after 7–8 days of incubation, exhibited brownish to black pigmentation from central and older region. *Termitomyces albuminosus* hyphal growth characters were as per standard pure *Termitomyces* cultural descriptions (Botha and Eicker 1991). The pigmentation radiated towards the margin (Figure 1(a,b)). Repeated subcultures of melanogenic strain produced same results. In shaken submerged condition, *T. albuminosus* culture consistently produced spiky brown to black pellets (Figure 1(c,d)). Melanin yield from *T. albuminosus* in present study was found to be 0.0142 ± 0.005 g/L from pelletized biomass.

### Melanin deposition sites and morphology of melanin granules

**Figure 2.**
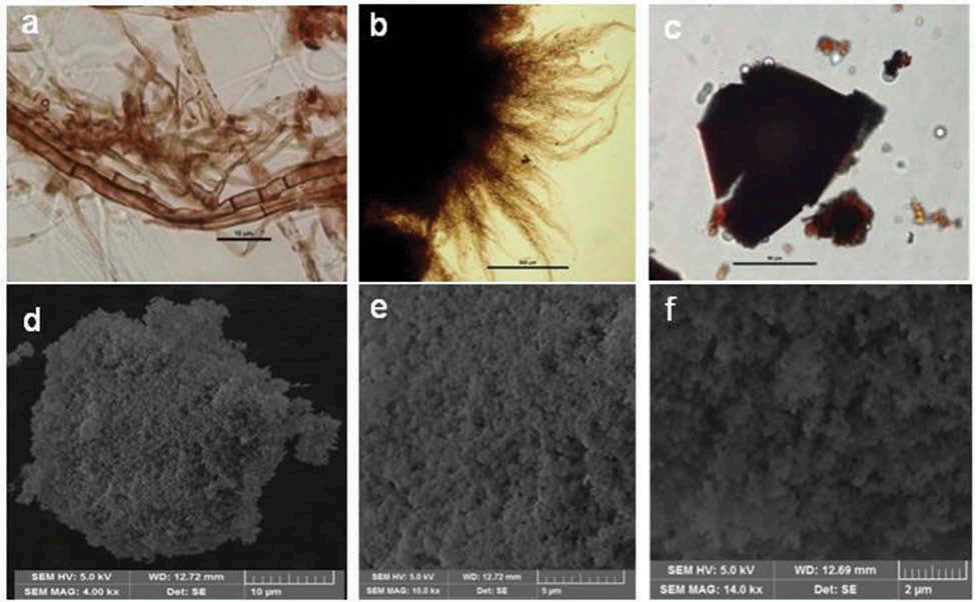
Microscopic analysis of *Termitomyces* melanin. (a) Cultural melanin with melanised hyphae showing cell wall bound and septal bound melanin under bright field view. (b) Pellet with spiky appendages cross section showing dark brown to black central core. (c) Pure dry melanin powder under bright field view. (d–f) Pure melanin granules at different magnifications under SEM view.

Micromorphologically *T. albuminosus* culture mat showed uniform deposition of brown–black pigment in hyphal cell wall and septa consistent with present knowledge (Figure 2(a)). Pellets showed central zone as dense black with brown peripheral spiky appendages (Figure 2(b)). Direct mount of purified melanin granules under bright field showed their polymorphic nature forming very thin, opaque amorphous black plates (Figure 2(c)). SEM images of purified sample showed the ultrafine structure of these thin amorphous plates comprising large clusters of almost spherical, compacted nanogranules. The plates show interesting but complex microtopography of nanogranules having 400–100 nm size (Figure 2(d,e,f)). Table 1 indicates the fractal analysis of pure melanin with fractal dimension *D* = 1.195–1.733.

**Table 1.** Fractal analysis of Melanin.

Fractal dimensions methods	Mean ± SD
Dilation	1.357 ± 0.050
Euclidean distance map	1.315 ± 0.048
Box counting	1.350 ± 0.081
Fast	1.155 ± 0.027
Fast (hybrid)	1.195 ± 0.034
Parallel lines	1.224 ± 0.032
Cumulative intersection	1.733 ± 0.084
Mass radius (long)	1.230 ± 0.051
Mass radius (short)	1.232 ± 0.050
Corner (count)	1.610 ± 0.078
Corner (perimeter)	1.616 ± 0.050

Note. Values are mean of (*n* = 3), ± SD (standard deviation).

### Elemental composition

Elemental analysis of *Termitomyces* melanin mainly indicated C:H:N:O:S composition percentage as 54.679%, 3.544%, 2.492%, 26.924%, and 12.361% as listed in Table 2. The sulphur content was not directly estimated due to lack of S detection probe but derived stoichiometrically which is an alternative method and presence of S was confirmed by the positive Lassaigne’s test.

**Table 2.** Elemental composition of melanin.

Sample	Content %				
	C	H	N	O	S
Pure *Termitomyces* *albuminosus* melanin	54.679	3.544	2.492	26.924	12.361

Note. The sulphur content was calculated from the equation (Harki et al. 1997). S%= (100)–(∑ C %+ H % + N %+ O %).

### UV -VIS and FTIR studies

**Figure 3.**
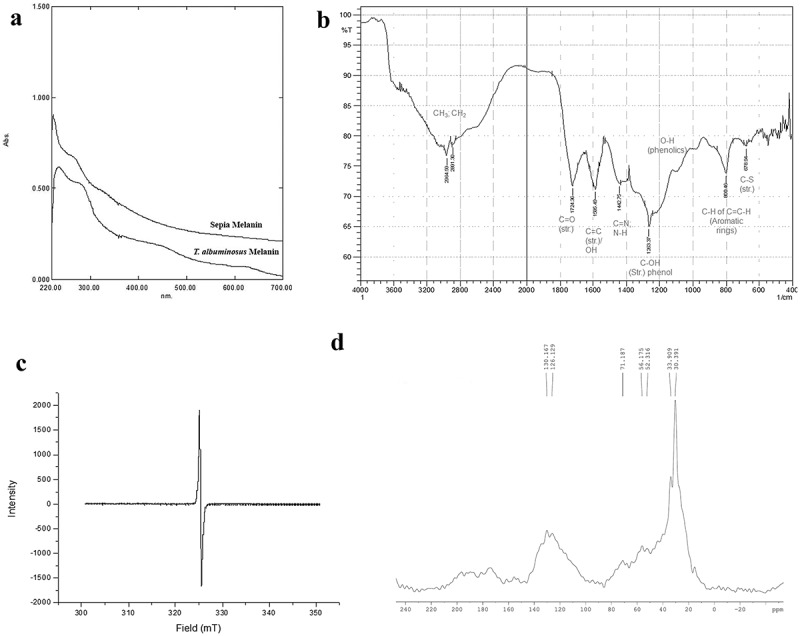
Spectral analysis of *Termitomyces albuminosus* melanin. (a) UV–VIS spectra of melanin. (b) FTIR spectrum of *T. albuminosus* melanin. (c) EPR of melanin. (d) ^13^C (CP/MAS) NMR spectra of melanin.

UV-VIS spectrum showed absorption profile identical to standard sepia melanin. The absorption spectra of *T. albuminosus* melanin showed characteristic peak in the ultraviolet region at 233 nm and not in visible region (Figure 3(a)). Melanin from *T. albuminosus* culture also produced a linear form with a negative slope of −0.0026.

The infrared spectrum of melanin exhibited absorption band at 2964 cm^−1^ and 2891 cm^−1^, indicating the presence of CH_3_, CH_2_ aliphatic group. The 1724 cm^−1^, 1585 cm^−1^ and 1442 cm^−1^ bands indicate C = O, C = C and C = N / N–H group, whereas 1263 cm^−1^ indicates phenolic C–O–H band (Figure 3(b)). Table 3 provides a comparative view of FTIR spectral band analysis of *T. albuminosus* melanin with other fungal melanins. *Termitomyces albuminosus* melanin showed characteristic bands for aromatic rings and sulphur at 800 cm^−1^ and 678 cm^−1^.

**Table 3.** FTIR spectroscopic characteristics of melanin.

Fungus	Bands (cm^−1^)	Assignments	References
*Phyllosticta capitalensis*	3352.5 1639.8	–OH, N–H bonds Conjugated carbonyl bonds	Suryanarayanan et al. (2004)
*Auricularia auricula*	1627.76 3422 2923.99 2853.83	Aromatic C=C & COO^−^group O–H stretching & NH_2_ groups Aliphathic group CH_3_ & CH_2_	Zhang et al. (2015)
*Pleurotus cystidiosus*	3445.05	OH group	Selvakumar et al. (2008)
*Auricularia auricula*	3287.6 2925.8 2851.2 1702.3 1619.4 1378.8	OH & NH group CH_3_ group CH_2_ group C=O & COO^–^ group	Bin et al. (2012)
*Termitomyces albuminosus*	2964 2891 1724 1585, 1442 1263 800 730, 710, 678	CH_3_ Aliphathic group CH_2_ group C=O stretching Overlapping O–H (def.) of C=C ring stretching C–O stretching due to phenol C–H (def.) of C=C–H (o.o.p.) from aromatic rings Weak absorption indicating C-S stretching	Present study

### EPR spectroscopy

In the present study, EPR spectrum showed the peak at 2.00968 (*G*-value) for *T. albuminosus* melanin (Figure 3(c)).

### NMR spectroscopy

^13^C (CP/MAS) NMR spectra are shown in Figure 3(d). Its spectral band assignments along with other reported melanins are summarised in Table 4. Characteristic chemical shift at 70–30 ppm representing =C–S and C–H carbon of open-chain aliphatic carbons present in cysteine/DOPA was observed in ^13^C NMR spectrum of *Termitomyces*.

**Table 4.** ^13^C NMR spectroscopic characteristics of melanin.

Source and type of melanin	Chemical shift range (ppm)	Possible assignments	References
*Oidiodendron tenuissimum, Trichoderma harzianum, Ulocladium atrum, Hendersonula toruloidea, Eurotium echinulatum*	220–160	Carboxyl/carbonyl groups	Knicker et al. (1995)
	160–140	Aromatic COR or CNR groups	
	140–110	Aromatic C–H carbons, guaiacyl C-2/C-6 Olefinic carbons	
	110–90	Anomeric carbon of carbohydrates, C-2/C-6 of Syringyl	
	90–60	Carbohydrate- derived structures (C-2 to C-5) in hexoses, C-2 of some amino acids & higher alcohols	
	60–45	Methoxyl groups, C-6 of carbohydrates, C-2 of most amino acids	
	45–0	Methylene groups in aliphatic rings & chains, methyl groups bound to carbon	
Dopa melanin	172	Carbonyl carbon	Duff et al. (1988)
	143, 118	Aromatic carbons	
	55, 35	Aliphatic carbons	
Melanoma melanin	173	Carbonyl carbon	
	125	Aromatic carbons	
	53,33	Aliphatic carbons	
Sepia melanin	173	Carbonyl carbon	
	140–110	Aromatic carbons	
	70–30	Aliphatic carbons	
Sepia melanin	200–160	Carbonyl carbon	Adhyaru et al. (2003)
	160–135	Aromatic & Indolic Cq (non-protonated)	
	135–90	Aromatic & Indolic CH (protonated)	
	95–10	Aliphatic carbons	
Sepia melanin Free acid (MFA)	200–160	Carbonyl carbon	
	165–135	Aromatic & Indolic Cq (non-protonated)	
	135–100	Aromatic & Indolic CH (protonated)	
	95–10, 50–0	Aliphatic carbons	
Sepia melanin	200–187, 167, 164	Carbonyl carbon	Hervé et al. (1994)
	147–110	Aromatic & ethylenic Cq (non-protonated)	
	131–127, 119–95	Aromatic & ethylenic CH (protonated)	
	75–15	Aliphatic carbons	
*T. albuminosus* melanin	200–170	Carbonyl carbon	Present study
	160–110	Aromatic carbons	
	45–40	=C–S	
	71, 56, 52, 33, 30	Aliphatic carbons in cysteine/DOPA	

## Discussion

This is first report on formation of a dark melanin like pigment in *Termitomyces* colonies, a phenomenon noticed in natural fruitbodies and confirmation of the pigment as melanin. Despite taxonomic knowledge about universal occurrence of dark pigmentation in *Termitomyces* fruitbodies, no attention has been paid to establish its chemical identity as melanin. In addition, no reports have been found on melanogenesis in pure cultures of *Termitomyces* species. This may be due to availability of very few pure cultures available in world culture collections for scientific community to work. In spite of 90 total taxa recorded pending systematic revision and found listed in Index Fungorum mycological database (www.indexfungorum.org) indicating high diversity of *Termitomyces* species in Asia and Africa, the catalogues in World Federation for culture collection have only 11 *Termitomyces* strains listed globally. This may be due to relative lack of interest in high-frequency culturing of wild-edible *Termitomyces* species or failure to get healthy fruitbodies and viable spores for isolating mycelial cultures. The present study overcame the problem by obtaining several mycelial cultures from different *Termitomyces* species and zeroing down on a stable melanogenic strain of *T. albuminosus* able to show excellent growth on solid medium as well as under submerged culture conditions. Previously (Siddiquee et al. 2012, 2015) reported dark grey to black colouration in *T. heimii* and *T. aurantiacus* culture grown on Potato Dextrose Agar medium after 7 days but failed to identify the melanogenesis process. Zhang et al. (2015) reported melanin from culture free filtrate of *Auricularia auricula* in submerged culture conditions yielding 0.124–0.558 g/L. However, Sun et al. (2016b) reported yield of 2.22 g/L melanin in culture filtrate of *A. auricula* in complete medium containing lactose, yeast extract, tyrosine, calcium chloride and sodium chloride, but not estimated melanin bound to cultural biomass. In the present study, the final product of melanin accounted for about 0.012% (w/w) of dry biomass. Relatively *T. albuminosus* strain used in the present study yielded less melanin probably due to choice of the medium, being a symbiotic mushroom or many other physiological parameters which need to tested in future.

In melanised fungi, pigment is known to be localised in the cell wall, in the outermost layer or embedded within the wall as granules, layered in fibrils, or bound to cell wall chitin (Butler and Day 1998). In this study, *Termitomyces* melanin was microscopically detected to be present in cell wall or septa. Nanoparticle nature of melanin has been studied (Beltrán-García et al. 2014) and our results are consistent with the same. Consistent with the latest development in understanding the properties of such complex surfaces in topological quantum chemistry it would be interesting to see whether melanin nanogranules could also be subjected to topochemical studies (Bradlyn et al. 2017; Fiete 2017) which might explain some interesting properties. Melanins fractal dimensions results clearly implying that assembly of melanin nanogranules may occur in fractal pattern (Bridelli 1998; Eom et al. 2017). It has been known that melanin purification steps lead to dehydration thus making the polymer more aggregated and it results in loss of capacity for physiological interactions (Nicolaus 1968; Prota 1992). The aggregated structure of melanin is postulated to prevent reactive oxygen species formation because photoactive residues are less exposed (Beltrán-García et al. 2014) however the function of *T. albuminosus* melanin may be more complex as it is a mutualistic species with hypogeal anamorph and epigeal teleomorph (Piearce 1987) .

Melanin produced by DHN pathway contains carbon and oxygen only, while the L-DOPA pathway melanins also contain nitrogen. Melanin synthesised via the L-DOPA pathway is referred to as eumelanin. DOPA melanins in presence of oxygen and tyrosinase are also known to undergo cysteinylation (incorporation of cysteine in the polymer). These melanins, red or yellow-coloured pigments are termed as pheomelanins initially synthesised just like eumelanins and contain sulphur (El-Naggar and El-Ewasy 2017). *Termitomyces* melanin could be a form of sulphur-rich pheomelanin as this group mainly consists of sulphur-containing benzothiazine and benzathiozol derivatives. Generally, pheomelanins or DOPA melanin chemically modified by amino acids such as cys–DOPA melanins are known to have approx. 9–16% sulphur content. These findings are in accordance with those reported by Harki et al. (1997; Costa et al. 2015; Sun et al. 2016b). According to Ye et al. (2014), about 14.83% sulphur content was determined by elemental analysis from *Lachnum* YM404 strain. Also the effect of medium composition on melanin composition is known. According to Bull (1970), in *Aspergillus nidulans* melanin pigment varied in composition with response to growth medium and the most significant finding was the widely varying nitrogen content of the melanin in response to the growth medium. Bull (1970) reported percentage composition of melanin in Czapek Dox Medium as C = 56.40%, H = 6.55%, and N = 3.92–1.78% (on addition of DOPA & Catechol), indicating that melanin composition can vary from medium to medium. High sulphur content of melanin in *Termitomyces* is possible due to availability of sulphur-containing amino acids and sulphite reductase enzymes. Previously, Alofe (1991; Botha and Eicker 1992; Ijeh et al. 2016; Sun et al. 2017) reported sulphur-containing (methionine, cysteine) amino acids from *Termitomyces umkowaani, T. sagittaeformis T. reticulatus, T. robustus*, and *T. microcarpus* fruitbodies. These amino-acid compositions vary from one geographic region to another. Laccase enzyme which is known to play a key role in biosynthesis of melanin has been also reported from *Termitomyces* (Bose et al. 2007; Gangwar et al. 2016). Rahmad et al. (2014) identified sulphite reductase enzyme from *T. heimii* which plays a key role in sulphur assimilation. Our results indicate that *Termitomyces* species may have efficient sulphur metabolism involving an unidentified pathway linked to O-acetylserine to form cysteine (Leustek et al. 2000; Kopriva and Koprivova 2003). According to Plonka and Grabacka (2006), the possible melanin synthesis pathway in *Termitomyces* using laccase enzyme and source of sulphur pool as amino acids can be written as

DOPA→DOPAquinone→CysteinylDOPA→1,4-Benzothiazinylalanine→pheomalanin.

which is required to be tested in future as the present study only aimed at the characterisation of melanin pigment from genus *Termitomyces*.

The linear decrease in the absorption with increasing wavelength was observed for *Termitomyces* melanin similar to that reported by (Zhang et al. 2015). Absorption peaks in UV regions occur due to the presence of many conjugated structures in melanin molecule (Ou-Yang et al. 2004). The log of optical density of a melanin solution when plotted against wavelength produces a linear curve with negative slopes. Such characteristic straight lines with negative slopes have been obtained from some melanogenic fungi such as *Phyllosticta capitalensis* and *Auricularia auricula* with slope ranging −0.0015 to −0.0030 (Ellis and Griffiths 1974; Suryanarayanan et al. 2004; Bin et al. 2012; Zhang et al. 2015). The slopes of linear plots are often used to identify melanins and matching spectral features in the present work confirms the identity of *T. albuminosus* melanin.

TFTIR studies carried out by Sava et al. (2001) reported that absorption is reduced at 3450 cm^−1^ and 1650 cm^−1^, after acid hydrolysis treatment undertaken during purification step due to formation of reactions between phenolic and carboxylic groups to form lactones. Also treatment with chloroform and ethyl acetate could have reduced absorption at 2900–2850 cm^−1^ in spectra.

Melanin polymers are known to have paramagnetic character and o-semiquinone free radical with spin (*S* = 1/2). These unpaired electrons of free radicals obey EPR effect (Pilawa et al. 2017). Enochs et al. (1993) described a standardised and effective test for the identification of melanin pigment by identifying the presence of stable population of organic free radical signal. The *G*-value of fungal melanin is reported to be 2.0012 (Selvakumar et al. 2008). *Termitomyces albuminosus* melanin *G*-value is found to be somewhat higher which could be due to O-semiquinone free radicals. Bin et al. (2012) also showed higher *G*-value of 2.0042 for *Auricularia auricula* melanin. It has been known that sulphur-containing radicals show high *G*-value (Bolman et al. 1970); therefore, incorporation of a sulphur-rich scaffold in melanin of *T. albuminosus* may result in a high *G*-value.

TAliphatic amine structural elements are proposed to arise in ^13^C NMR spectrum from coupling of dopamine/quinone structural units which are unique to dopamine melanins (Della Vecchia et al. 2013; Chatterjee et al. 2014). Tian et al. (2003) reported that carbon-near sulphur shows chemical shift at 45–40 ppm and CH_2_ carbon–CH_2_–CH(NH_2_)–COOH of tyrosine/DOPA can also be seen around 40–35 ppm in ^13^C NMR spectrum which is consistent with our sulphur-containing melanin claim.

## Conclusions

The present study successfully established the chemical identity of the dark pigment as a unique form of fungal melanin with high sulphur content. The exact structure of melanin polymers is difficult to elucidate and the benefit of incorporation of a sulphur scaffold in *Termitomyces* melanin needs further exploration as it may play functionally important roles at crucial and critical stages in the natural life cycle of *Termitomyces* holomorph in protecting the species from injury and damage.
